# Supplementary material for: A reduced ability to discriminate social from non-social touch at the circuit level may underlie social avoidance in autism
Source: Nat Commun. 2025 May 17;16:4600. doi: 10.1038/s41467-025-59852-6 (PMC12085590; doi:10.1038/s41467-025-59852-6)
Supplement: Supplementary file 1 — Supplementary Information [file 41467_2025_59852_MOESM1_ESM.pdf]

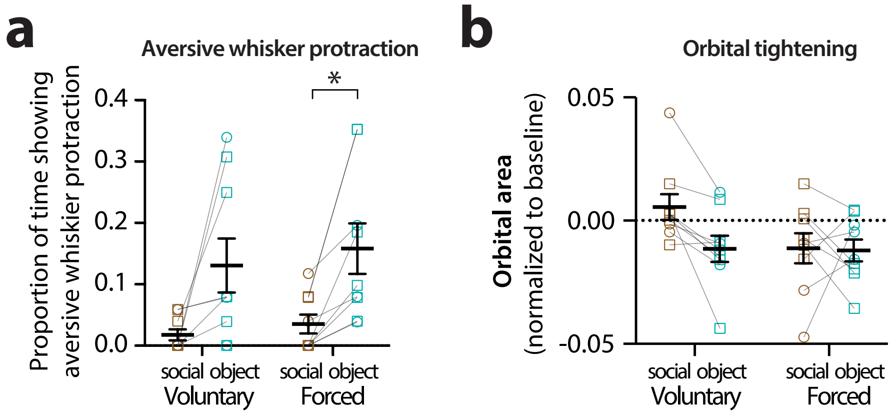

Relates to Fig. 1d

Decoding touch context (social vs. object) based on DLC labels (panels d-f)

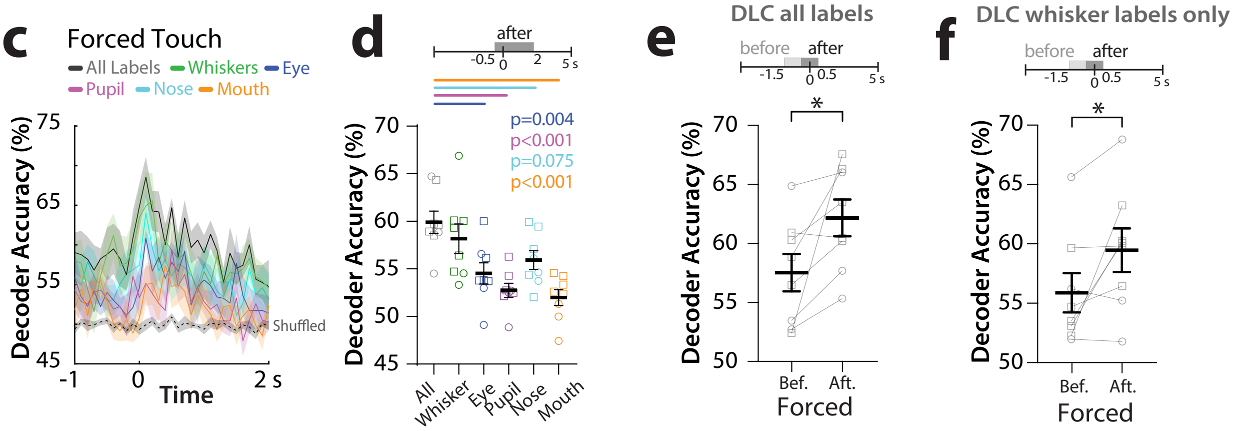

Relates to Fig. 1f Relates to Fig. 1g Relates to Fig. 1h Relates to Fig. 1k

Decoding touch choice (volunt. vs. forced)

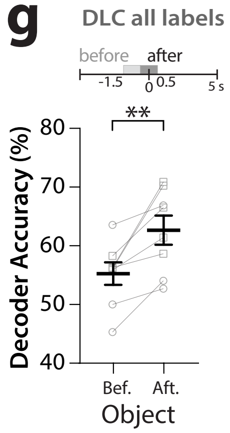

Relates to Fig. 1m

Supplementary Fig. 1: WT mice display aversive whisker protraction to forced object touch but not social touch.

a. Proportion of time that WT mice manifest sustained aversive whisker protraction in response social touch (brown) or object touch (cyan), over the first 5 voluntary or forced interactions. Squares=males, circles females. *$p<0.05$, two-way ANOVA with Bonferroni's.

b. Proportion of time that WT mice manifest sustained orbital area in response social touch (brown) or object touch (cyan), over the first 5 voluntary or forced interactions. Squares=males, circles females.

c. Decoder performance of touch context (social vs. object) using DLC labels on the face in WT mice across time (from 1 s before to 2 s after platform stops) for forced touch.

d. Decoder accuracy for context discrimination for using DLC labels for all facial features or labels for individual facial features (whiskers, eye, pupil, nose or mouth) during the time after platform movement (-0.5 s to +2 s) for forced touch. $p<0.01$ for nonparametric Kruskal-Wallis test.

e. Decoder accuracy for context discrimination using all DLC labels before (-1.5 to -0.5 s) and after (-0.5 to +0.5 s) platform movement for forced touch. *$p<0.05$ for parametric paired t-test.

f. Decoder accuracy for context discrimination using DLC whisker labels before (-1.5 to -0.5 s) and after (-0.5 to +0.5 s) platform movement for forced touch.

g. Decoder accuracy for choice discrimination using all DLC labels before (-1.5 to -0.5 s) and after (-0.5 to +0.5 s) platform movement for object touch. **$p<0.01$ for parametric paired t-test for both panels. N=9 mice for panels a-b, 8 mice for panels c-g.

All data presented as mean ± SEM.

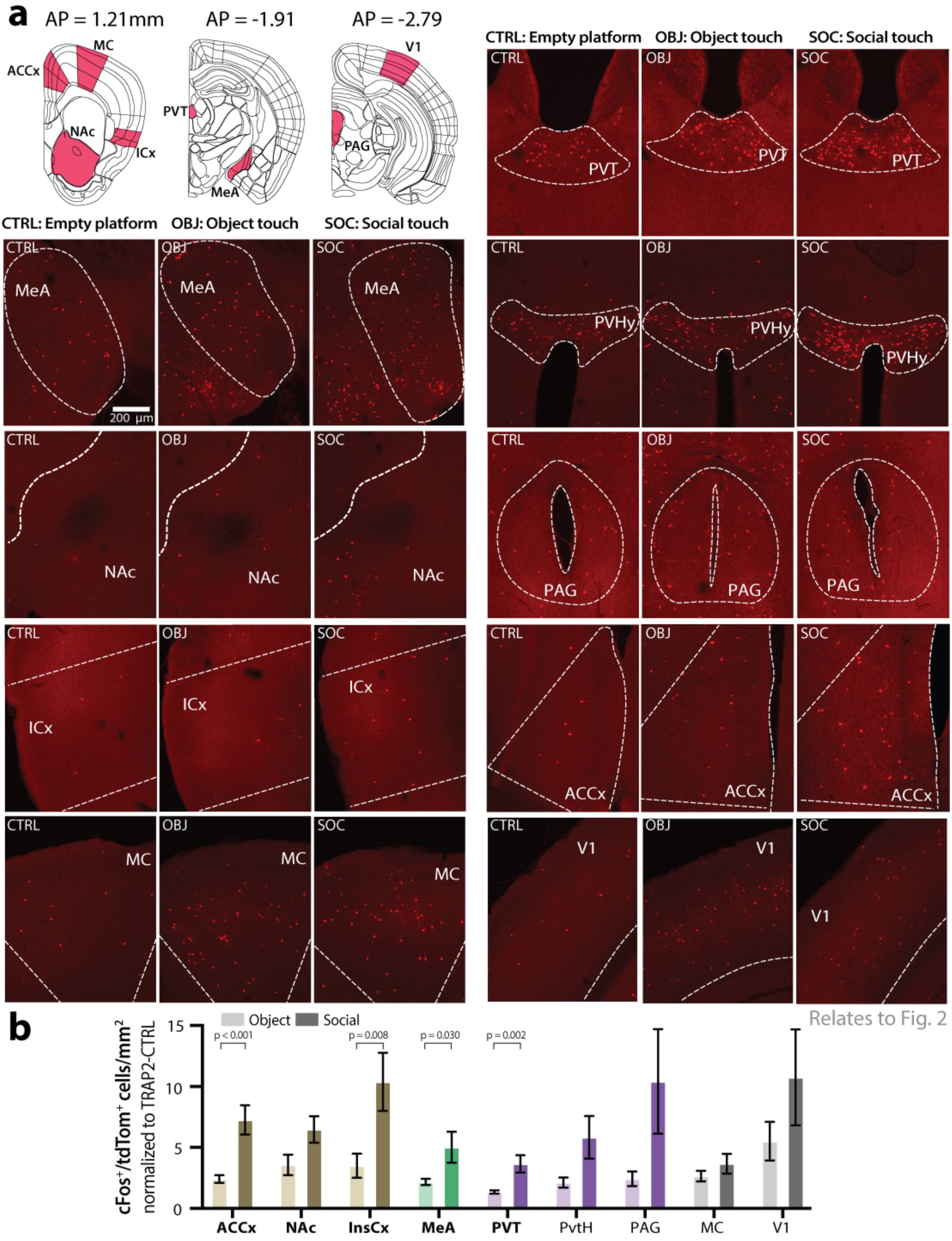

Relates to Fig. 2

Supplementary Fig. 2: Differential cFos expression to object touch vs. social touch across relevant brain regions.

a. Cartoons to indicate the locations across the anterior-posterior (AP) axis of the mouse brain where images of cFos expression in TRAP2 mice were taken (top left). Example images of cFos expression from medial amygdala (MeA), nucleus accumbens (NAc), insular cortex (InsCx), motor cortex (MC), paraventricular nucleus of the thalamus (PVT), paraventricular nucleus of the hypothalamus (PVHy), periaqueductal gray (PAG), anterior cinculate cortex (ACCx), and primary visual cortex (V1) during object touch (OBJ) and social touch (SOC), compared to the no touch (empty platform) control (CTRL).

b. Density of cFos-expressing (tdTom+) cells per mm^2 for mice that received repeated presentations of OBJ or SOC touch, normalized to the CTRL cell density for each brain region (bottom). ***$p<0.001$, **$p<0.01$, *$p<0.05$, normality was tested with D'Agostino & Pearson test followed by unpaired nonparametric Mann-Whitney or parametric t-test for each brain region. Each bar represents data from 5-6 mice, and at least 6 images were collected from a single mouse for each brain region. For object touch, n=33 ACCx, n=16 NAc, n=28 InsCx & n=25 MeA, n=30 PVT, n=16 PvtH, n=28 PAG, n=28 MC, n=23 V1 sections. For social touch, n=39 ACCx, n=19 NAc, n=26 InsCx & n=24 MeA, n=28 PVT, n=18 PvtH, n=23 PAG, n=23 MC, n=21 V1 sections. Data presented as mean ± SEM.

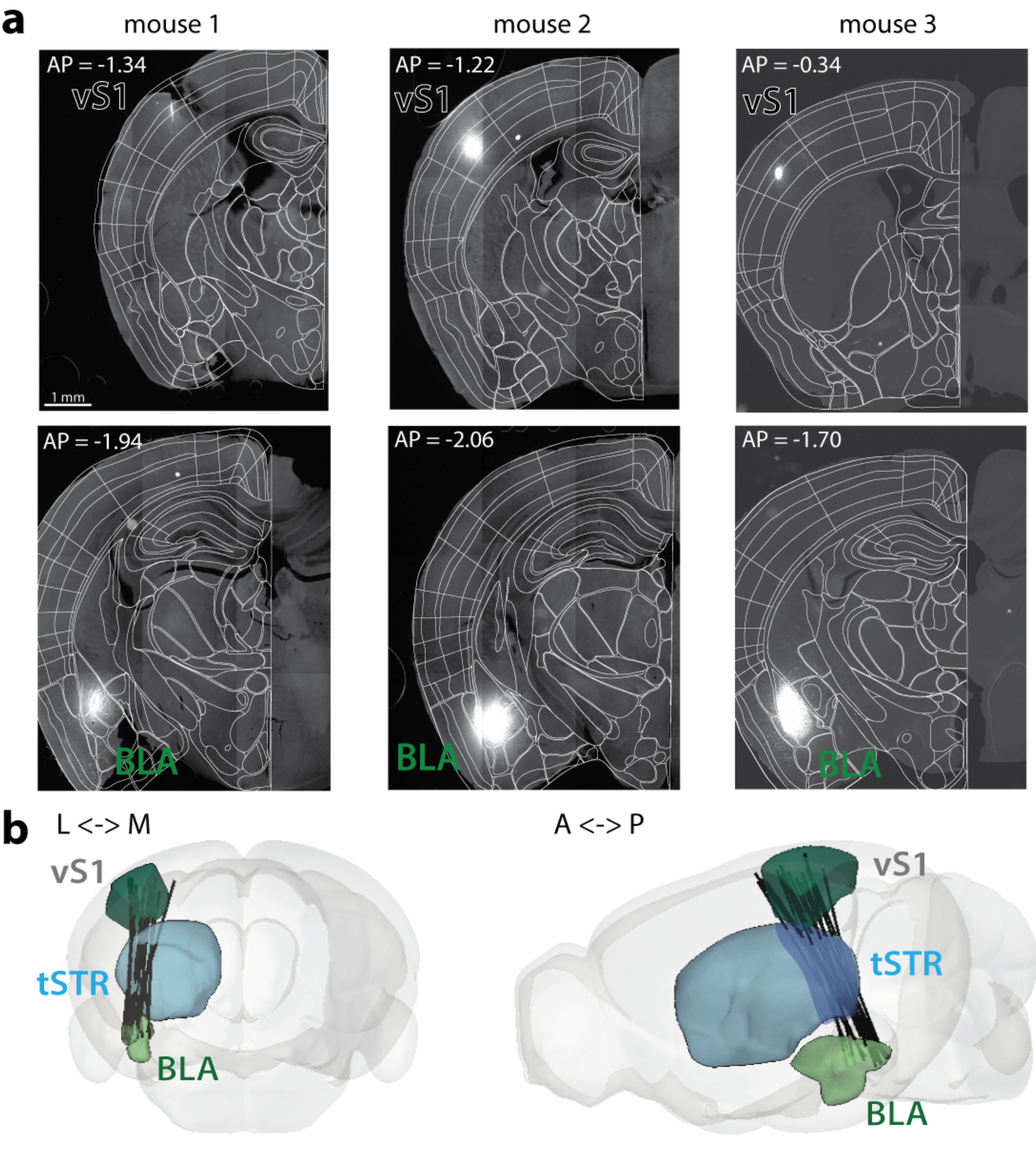

Relates to Fig. 3

Supplementary Fig. 3: Histological reconstruction of the trajectory of Neuropixels probes.

a. Example fluorescence imaging in three mouse brains of the probe shank trajectory (stained with DiI) to confirm accurate targeting of vS1, tSTR and BLA. Brain sections (50 µm thickness) are aligned to the Allen Mouse Brain Atlas (scale bar = 1 mm).

b. 3D reconstruction views of Neuropixels probe tract across the brains of all WT (n=9) and *Fmr1* KO mice (n=10) using the location of DiI fluorescence in coronal brain slices. We confirmed that each probe traverses vS1, tSTR and BLA across the mediolateral (M-L) and anteroposterior (A-P) axes.

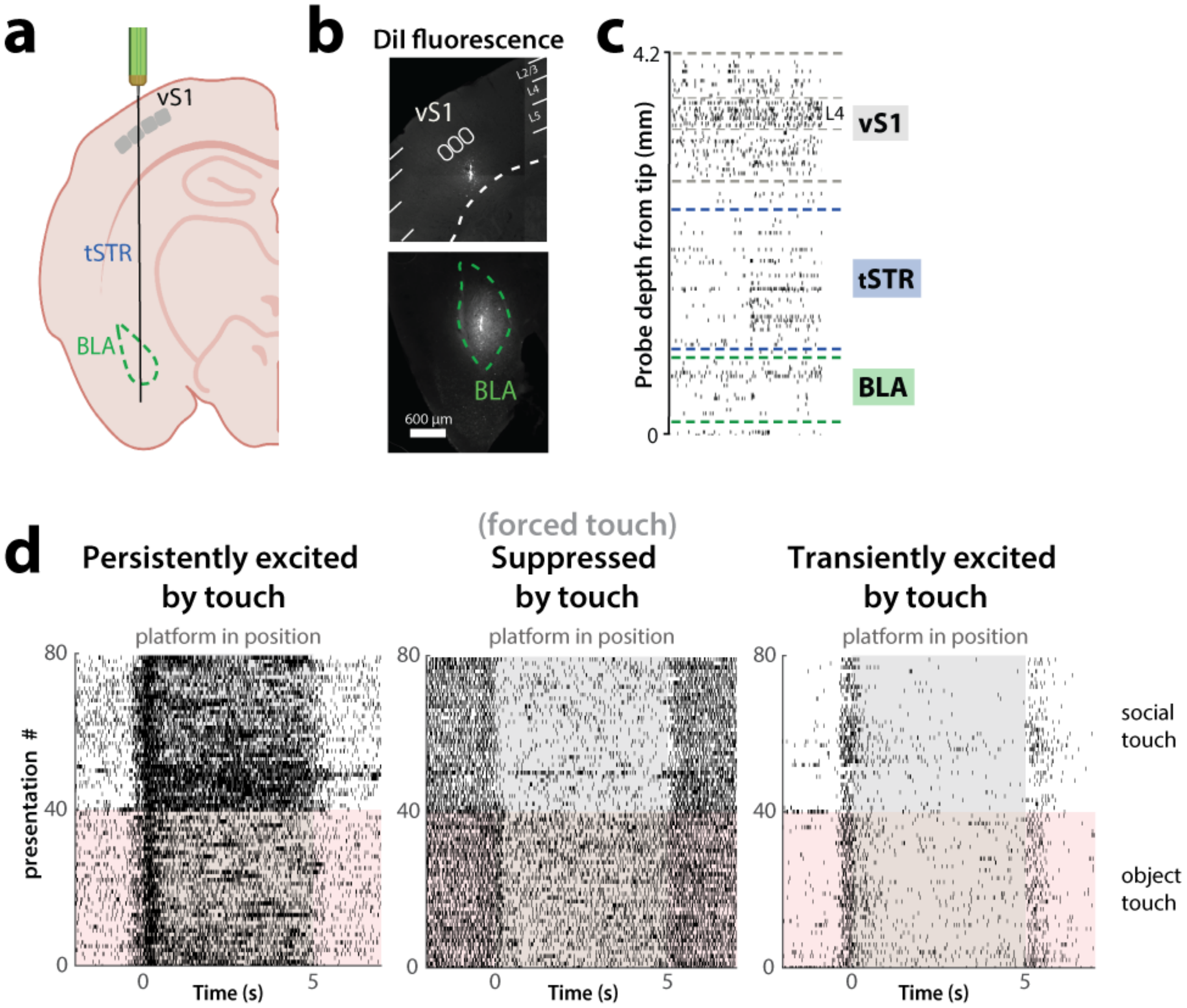

Relates to Fig. 3

Supplementary Fig. 4: Approach to allocate individual units to vS1, tSTR or BLA.

a. Cartoon showing Neuropixels probe implanted at 0° angle using mouse brain coordinates -1.46 AP, 2.9 ML 3.75 DV to target vS1, tSTR and BLA.

b. DiI fluorescence used to confirm probe targeting in vS1 and BLA (scale bar=600 μm).

c. Patterns of action potential spiking across time and depth (in mm) were used to allocate units recorded from Neuropixels to different brain regions, in addition to the estimated depth of the unit inferred from the probe trajectory reconstructions.

d. Example rasters of all action potential spikes across all 40 forced presentations of social touch (top) and object touch (bottom) for three example units that are persistently excited by touch, suppressed by touch, or transiently excited by the initial contact just before platform stops. Time 0 s denotes when the platform stops moving towards the test mouse and 5 s denotes when the platform starts moving away from the test mouse. Note how the left-most unit is preferentially excited by social touch, while the middle unit is preferentially suppressed by social touch.

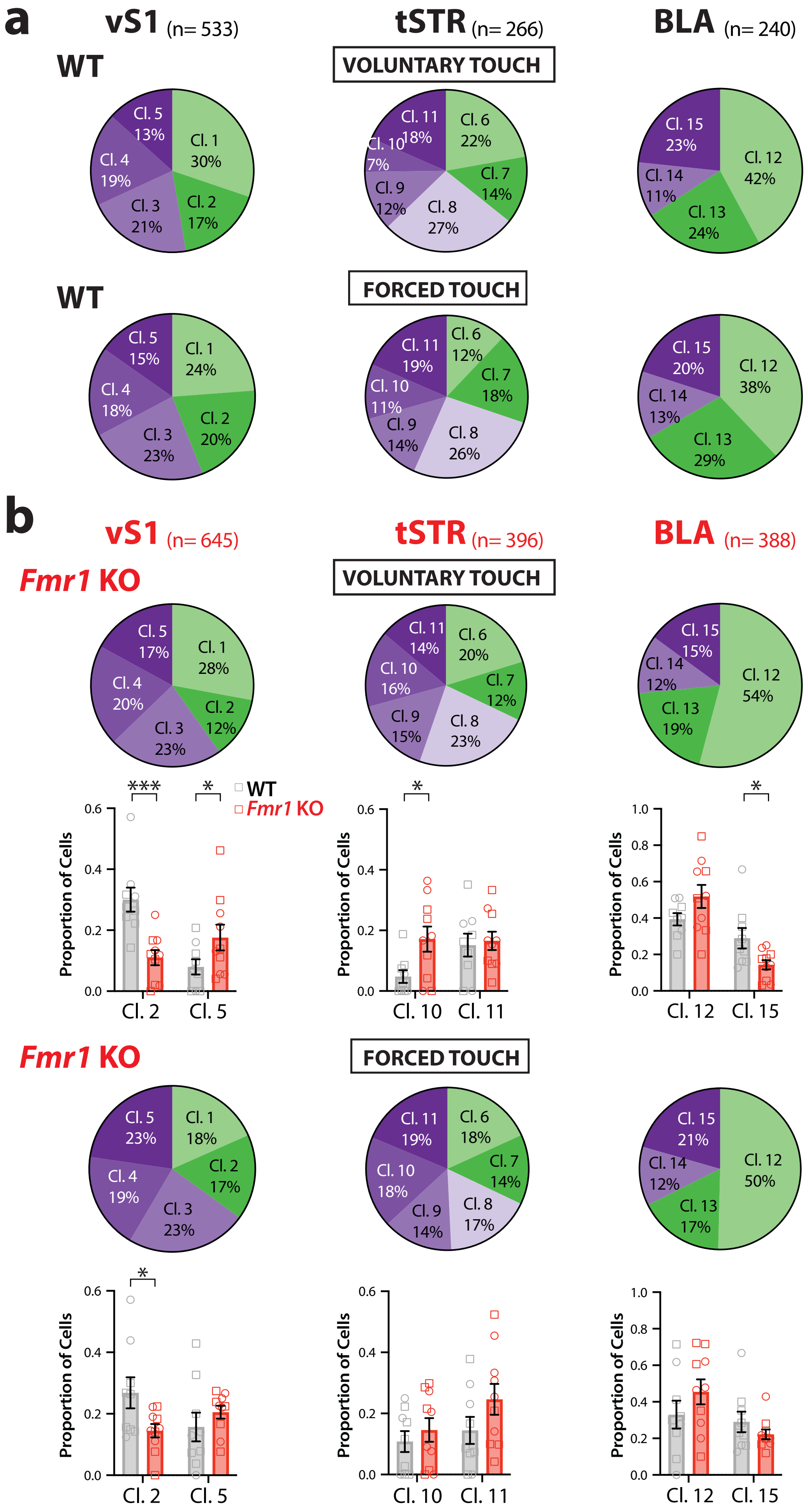

a
vS1 (n= 533)
tSTR (n= 266)
BLA (n= 240)
WT
VOLUNTARY TOUCH
Cl. 5 13%
Cl. 1 30%
Cl. 4 19%
Cl. 3 21%
Cl. 2 17%
Cl. 11 18%
Cl. 6 22%
Cl. 10 7%
Cl. 7 14%
Cl. 9 12%
Cl. 8 27%
Cl. 15 23%
Cl. 12 42%
Cl. 14 11%
Cl. 13 24%
WT
FORCED TOUCH
Cl. 5 15%
Cl. 1 24%
Cl. 4 18%
Cl. 2 20%
Cl. 3 23%
Cl. 11 19%
Cl. 6 12%
Cl. 7 18%
Cl. 10 11%
Cl. 9 14%
Cl. 8 26%
Cl. 15 20%
Cl. 12 38%
Cl. 14 13%
Cl. 13 29%
b
vS1 (n= 645)
tSTR (n= 396)
BLA (n= 388)
Fmr1 KO
VOLUNTARY TOUCH
Cl. 5 17%
Cl. 1 28%
Cl. 4 20%
Cl. 2 12%
Cl. 3 23%
Cl. 11 14%
Cl. 6 20%
Cl. 10 16%
Cl. 7 12%
Cl. 9 15%
Cl. 8 23%
Cl. 15 15%
Cl. 14 12%
Cl. 12 54%
Cl. 13 19%
WT
Fmr1 KO
Proportion of Cells
Cl. 2
Cl. 5
Cl. 10
Cl. 11
Cl. 12
Cl. 15
Fmr1 KO
FORCED TOUCH
Cl. 5 23%
Cl. 1 18%
Cl. 2 17%
Cl. 4 19%
Cl. 3 23%
Cl. 11 19%
Cl. 6 18%
Cl. 7 14%
Cl. 10 18%
Cl. 9 14%
Cl. 8 17%
Cl. 15 21%
Cl. 12 50%
Cl. 14 12%
Cl. 13 17%
Proportion of Cells
Cl. 2
Cl. 5
Cl. 10
Cl. 11
Cl. 12
Cl. 15

Supplementary Fig. 5: Proportion of total cells in each cluster differs within WT and between WT and *Fmr1* KO mice.

a. Percentage of cells belonging to different clusters in vS1, tSTR and BLA for all WT mice (n=9) during voluntary touch (top) or forced touch (bottom).

b. Percentage of cells belonging to different clusters for all *Fmr1* KO mice (n=10) (top). Quantification of the relative abundance of cells in various clusters (as a proportion of total cells) for different WT and *Fmr1* KO mice during voluntary and forced touch (bottom). ***p<0.001, *p<0.05 for unpaired parametric t-test WT vs *Fmr1* KO for each cluster. Squares=males, circles=females.

Data is presented as mean ± SEM.

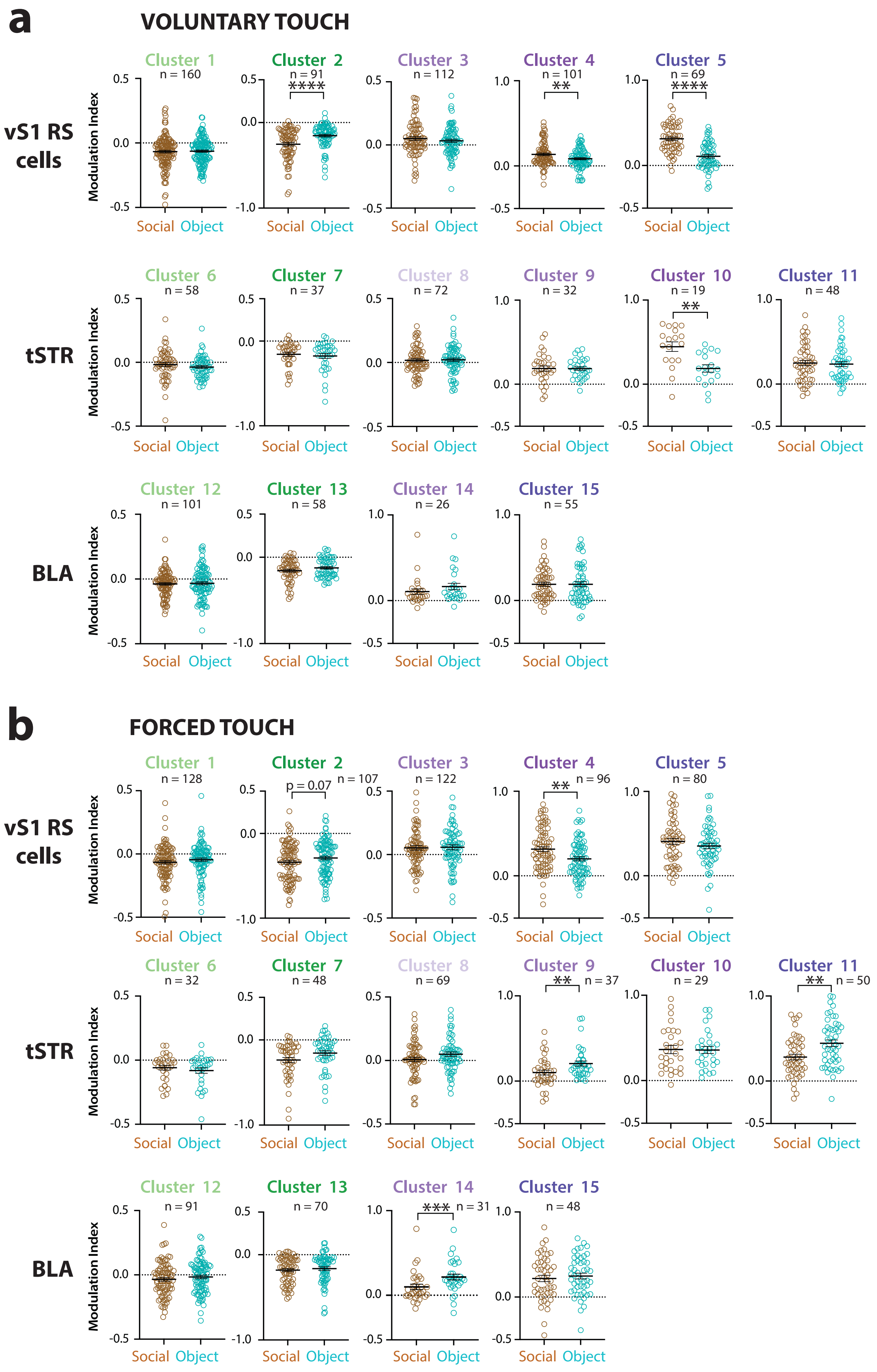
a
VOLUNTARY TOUCH
vS1 RS cells
tSTR
BLA
Modulation Index
Cluster 1
Cluster 2
Cluster 3
Cluster 4
Cluster 5
Cluster 6
Cluster 7
Cluster 8
Cluster 9
Cluster 10
Cluster 11
Cluster 12
Cluster 13
Cluster 14
Cluster 15
Social Object
b
FORCED TOUCH
p = 0.07

Supplementary Fig. 6: Clusters in vS1, tSTR and BLA are modulated differently by voluntary social and object touch in WT mice.

a. Modulation index of vS1 RS, tSTR and BLA cells in different clusters for social vs. object touch, during voluntary presentations, as an average of all 40 presentations. N denotes number of cells for the cluster.

b. Same as in panel *a*, but for forced touch. ****p<0.001, ***p<0.005, **p<0.01 for paired parametric t-test. Each symbol represents a single cell taken from across 9 WT mice.

Data presented as mean ± SEM.

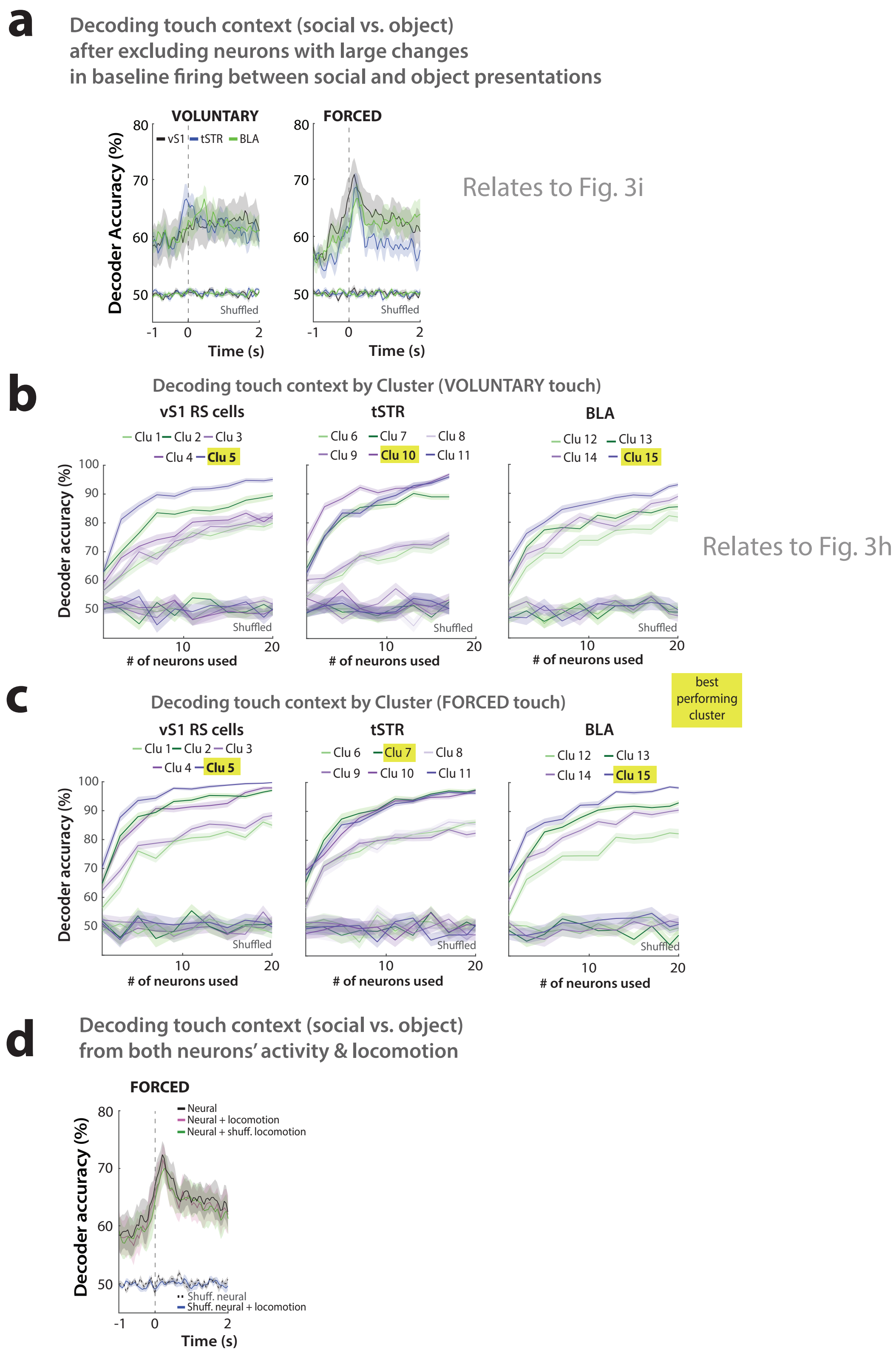
a
Decoding touch context (social vs. object)
after excluding neurons with large changes
in baseline firing between social and object presentations
VOLUNTARY
FORCED
vS1
tSTR
BLA
Decoder Accuracy (%)
80
70
60
50
Shuffled
-1
0
2
Time (s)
Relates to Fig. 3i
b
Decoding touch context by Cluster (VOLUNTARY touch)
vS1 RS cells
Clu 1
Clu 2
Clu 3
Clu 4
Clu 5
tSTR
Clu 6
Clu 7
Clu 8
Clu 9
Clu 10
Clu 11
BLA
Clu 12
Clu 13
Clu 14
Clu 15
Decoder accuracy (%)
100
90
80
70
60
50
Shuffled
10
20
# of neurons used
Relates to Fig. 3h
best performing cluster
c
Decoding touch context by Cluster (FORCED touch)
vS1 RS cells
Clu 1
Clu 2
Clu 3
Clu 4
Clu 5
tSTR
Clu 6
Clu 7
Clu 8
Clu 9
Clu 10
Clu 11
BLA
Clu 12
Clu 13
Clu 14
Clu 15
Decoder accuracy (%)
100
90
80
70
60
50
Shuffled
10
20
# of neurons used
d
Decoding touch context (social vs. object)
from both neurons' activity & locomotion
FORCED
Neural
Neural + locomotion
Neural + shuff. locomotion
Decoder accuracy (%)
80
70
60
50
Shuff. neural
Shuff. neural + locomotion
-1
0
2
Time (s)

Supplementary Fig. 7: Decoding touch context (social vs. object) from mean neural activity in vS1, tSTR and BLA.

a. Decoder accuracy for touch context based on activity of 10 randomly selected cells from each mouse in vS1, tSTR and BLA for every 50 ms during the stimulation period of forced touch (-2, 7s) after excluding neurons with large changes in baseline firing between social and object presentations. For voluntary touch, 9.2% of vS1, 9.4% of tSTR and 8.8% of BLA cells were excluded. For forced touch, 7.8% of vS1, 9.1% of tSTR and 8.3% of BLA cells were excluded.

b. Decoder accuracy for touch context based on the activity of cells in vS1, tSTR and BLA during voluntary touch (0 to 5 s). From each brain region, 1-20 neurons were randomly selected to be used in the SVM classifier. Decoder accuracy is also shown for shuffled data, where context identity was shuffled in 80% of object and social touch presentations (64 stimulations total) used for the training data set.

c. Decoder accuracy for touch context based on the activity of cells in vS1, tSTR and BLA during forced touch (0 to 5 s). From each brain region, 1-20 neurons were randomly selected to be used in the SVM classifier. Decoder accuracy is also shown for shuffled data, where context identity was shuffled in 80% of object and social touch presentations (64 stimulations total) used for the training data set.

d. Decoder accuracy for touch context based on activity (or shuffled activity) of 10 randomly selected cells and including locomotion (or shuffled locomotion) from each mouse in vS1, tSTR and BLA for every 50 ms during the stimulation period of forced touch (-1, 2s).

Line and shaded area correspond to mean ± SEM.

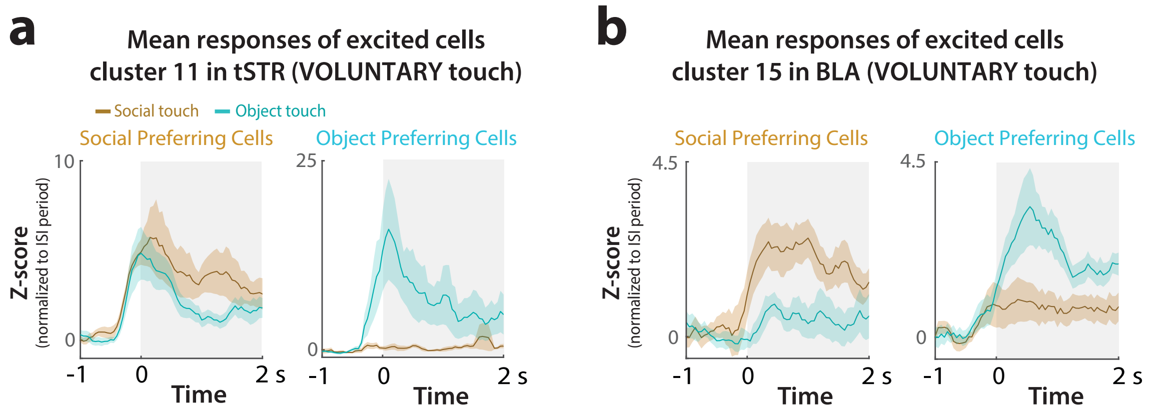

Relates to Fig. 4c

Supplementary Fig. 8: Responses of social-preferring and object-preferring cells in tSTR and BLA.

a. Averaged z-score firing rate of all social and object preferring cells in Cl. 11 of the tSTR during voluntary object and social touch.

b. Just as in panel *a* but for cells in Cl. 15 of the BLA. Data presented as mean ± SEM.

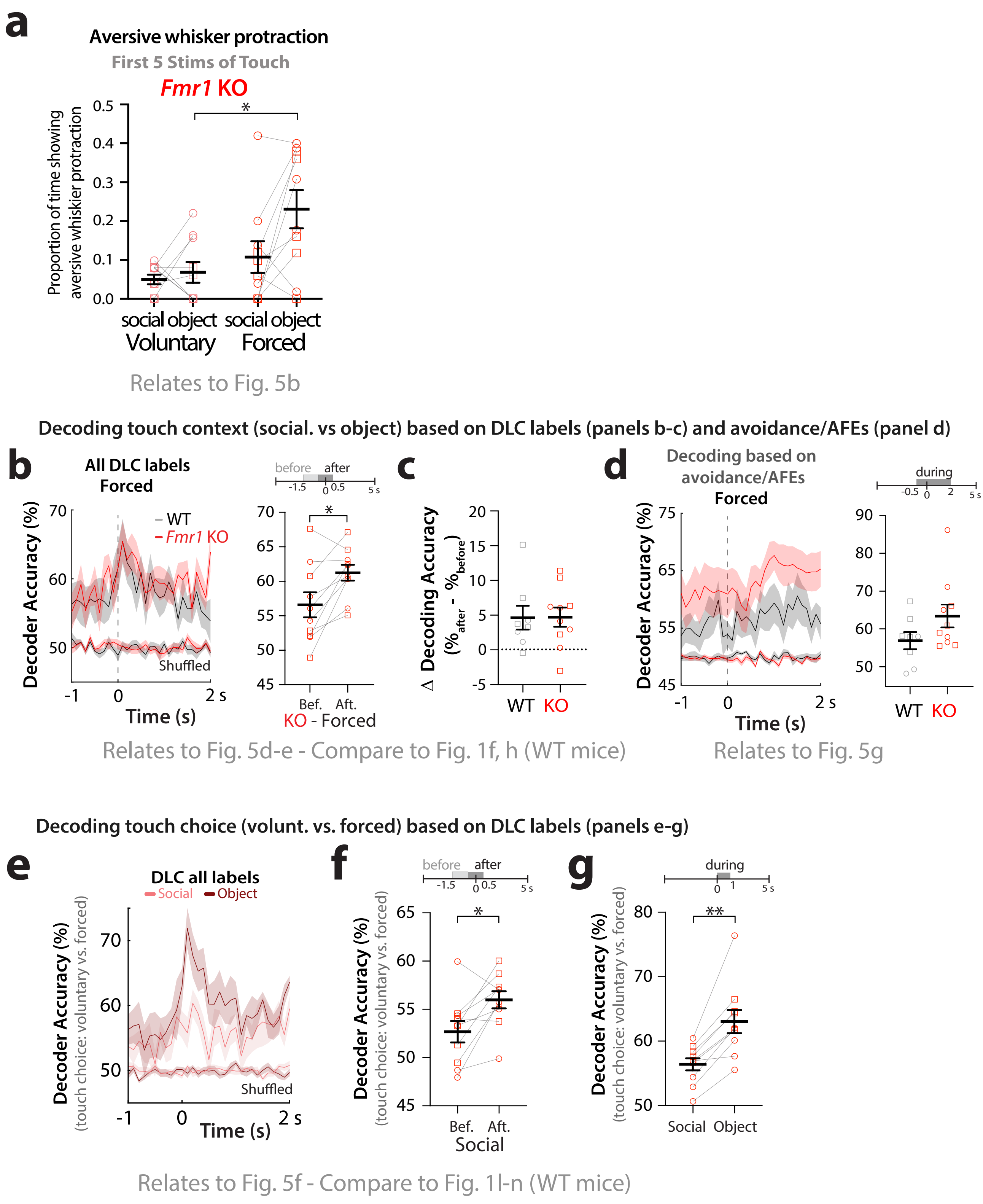

a
Aversive whisker protraction
First 5 Stims of Touch
Fmr1 KO
Proportion of time showing aversive whisker protraction
social object social object
Voluntary Forced
Relates to Fig. 5b
Decoding touch context (social. vs object) based on DLC labels (panels b-c) and avoidance/AFEs (panel d)
b
All DLC labels
Forced
Decoder Accuracy (%)
WT
Fmr1 KO
Shuffled
Time (s)
before after
Bef. Aft.
KO - Forced
c
Δ Decoding Accuracy (%after - %before)
WT KO
d
Decoding based on avoidance/AFEs
Forced
during
Relates to Fig. 5d-e - Compare to Fig. 1f, h (WT mice)
Relates to Fig. 5g
Decoding touch choice (volunt. vs. forced) based on DLC labels (panels e-g)
e
DLC all labels
Social
Object
Decoder Accuracy (%)
(touch choice: voluntary vs. forced)
Shuffled
Time (s)
f
Bef. Aft.
Social
g
Social Object
Relates to Fig. 5f - Compare to Fig. 1l-n (WT mice)

Supplementary Fig. 9: *Fmr1* KO mice show similar levels of aversive whisker protraction for social touch and object touch and find social touch within their personal space more aversive.

a. Proportion of time that *Fmr1* KO mice exhibit sustained aversive whisker protraction during social touch and object touch for voluntary and forced presentations. Squares=males, circles=females. *p<0.05 for two-way ANOVA with Bonferroni's.

b. Left: Decoding touch context (social vs. object) using all DLC labels during forced touch interactions for WT and *Fmr1* KO mice from -1 s to +2 s after platform stops (left) right: quantification of decoding accuracy before (-1.5 to -0.5 s) and after (-0.5 to 0.5 s) platform stops.

c. Change in decoder accuracy (% after minus % before) for touch context using all DLC whisker labels during forced touch interactions in *Fmr1* KO and WT.

d. Like panel b, but decoding touch context based on running avoidance and AFEs during forced touch interactions for WT and *Fmr1* KO mice.

e. Decoder performance for touch choice (voluntary vs. forced) discrimination in *Fmr1* KO mice using SVM classifiers trained on all DeepLabCut (DLC) labels on the mouse's face across time for social touch and object touch (from -1 s to +2 s after platform stops).

f. Decoder accuracy for touch choice discrimination in *Fmr1* KO mice using all DLC labels before (-1.5 to -0.5 s) and after (-0.5 to 0.5 s) platform stops (left). *p<0.05.

g. Decoder accuracy for touch choice discrimination in *Fmr1* KO mice using all DLC labels during the first second after platform stops for social touch and object touch (right). **p<0.01 for parametric paired t-test for both panels. N=10 mice for panels a-b, e-g. N=8 WT and 10 *Fmr1* KO mice for panels c-d.

All data presented as mean ± SEM.

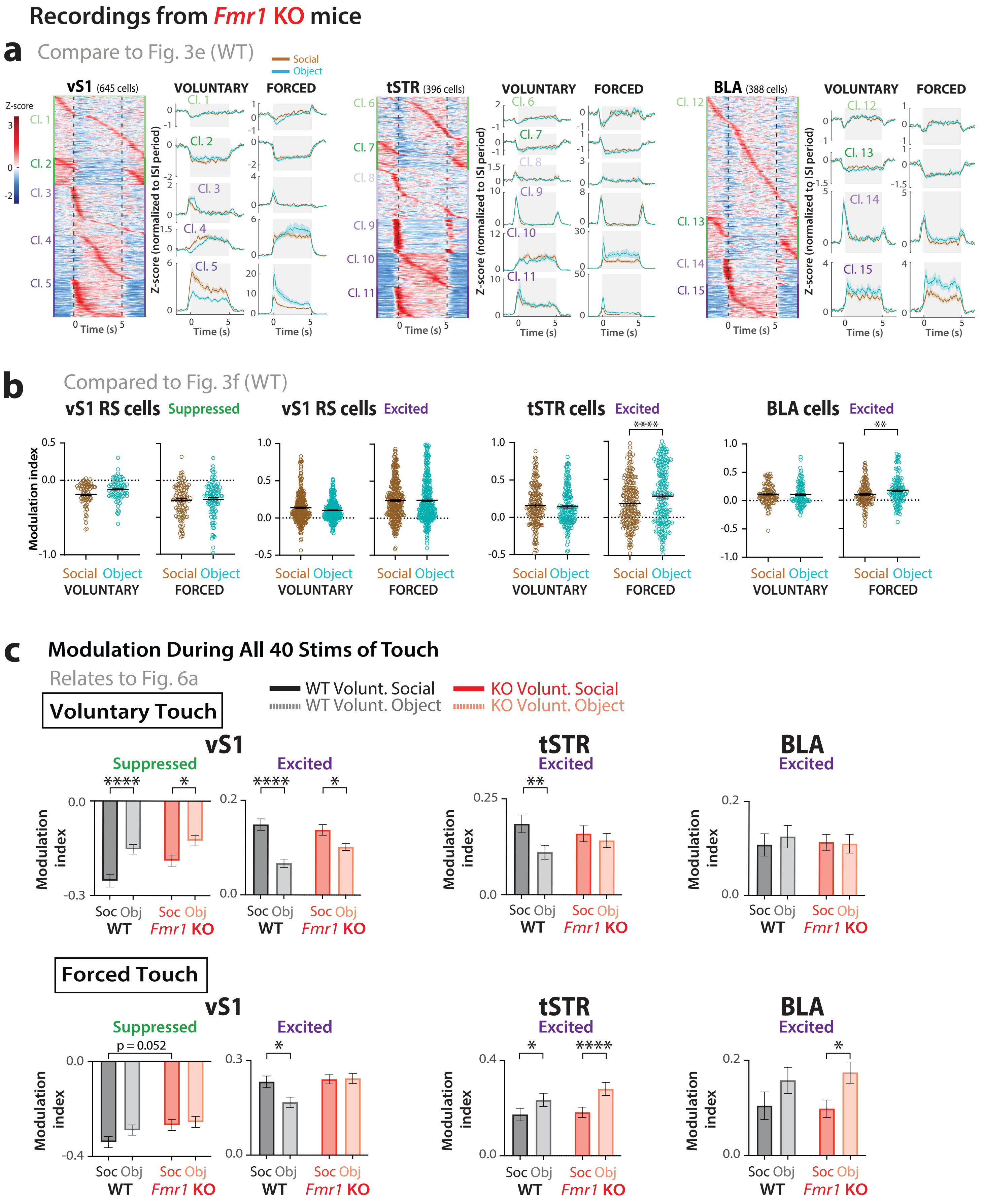

Recordings from Fmr1 KO mice
a
Compare to Fig. 3e (WT)
Social
Object
vS1 (645 cells)
VOLUNTARY
FORCED
tSTR (396 cells)
BLA (388 cells)
Z-score
Cl. 1
Cl. 2
Cl. 3
Cl. 4
Cl. 5
Cl. 6
Cl. 7
Cl. 8
Cl. 9
Cl. 10
Cl. 11
Cl. 12
Cl. 13
Cl. 14
Cl. 15
Z-score (normalized to ISI period)
Time (s)
b
Compared to Fig. 3f (WT)
vS1 RS cells Suppressed
vS1 RS cells Excited
tSTR cells Excited
BLA cells Excited
Modulation index
Social Object
VOLUNTARY
FORCED
c
Modulation During All 40 Stims of Touch
Relates to Fig. 6a
Voluntary Touch
WT Volunt. Social
WT Volunt. Object
KO Volunt. Social
KO Volunt. Object
vS1
tSTR
BLA
Suppressed
Excited
Modulation index
Soc Obj
WT
Fmr1 KO
Forced Touch
p = 0.052

Supplementary Fig. 10: Clusters in vS1, tSTR and BLA are modulated differently by voluntary social and object touch in *Fmr1* KO mice.

a. Left: Heatmap of the trial-averaged PSTHs for voluntary touch (taken as an average of all object and social touch presentations) for all vS1, tSTR and BLA cells split by clusters derived from PCA-k-means clustering and sorted by peak firing in time within each cluster. Right: Mean Z-scores for all neurons of individual clusters for social vs. object touch during voluntary or forced presentations, as a mean of all 40 presentations. Clusters are sorted by suppressed (green) to excited (purple) and shading indicates magnitude of how much they were suppressed/excited. Time 0 s denotes when the platform stops in the contact position.

b. Modulation index of vS1 RS excited and suppressed cells, tSTR excited cells, and BLA excited cells for social vs. object touch**.** ****p<0.0001, **p<0.01 for paired parametric t-test. Each symbol represents a single cell (N=10 *Fmr1* KO mice).

c. Modulation index of vS1 RS cells excited and suppressed by touch, as well as excited cells in tSTR and BLA in response to voluntary (top) and forced (bottom) social vs. object touch, as an average of all 40 stimulations, in WT and *Fmr1* KO mice. ****p<0.001, **p<0.01, *p<0.05 for two-way ANOVA with Bonferroni's. For WT excited cells, vS1 n=227/220, tSTR 99/116, and BLA 81/79. For *Fmr1* KO excited cells, vS1 n=307/331, tSTR 178/202, and BLA 105/128. For *Fmr1* KO, suppressed vS1 n=77/110 (panels b-c).

All data presented as mean ± SEM.

Comparing mean firing rates in WT mice and *Fmr1* KO mice   Relates to Fig. 6

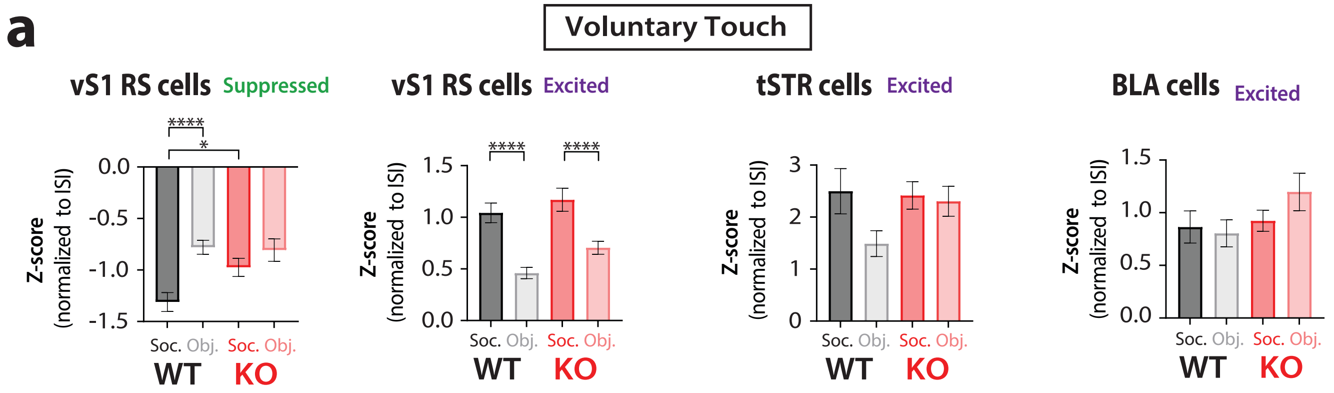

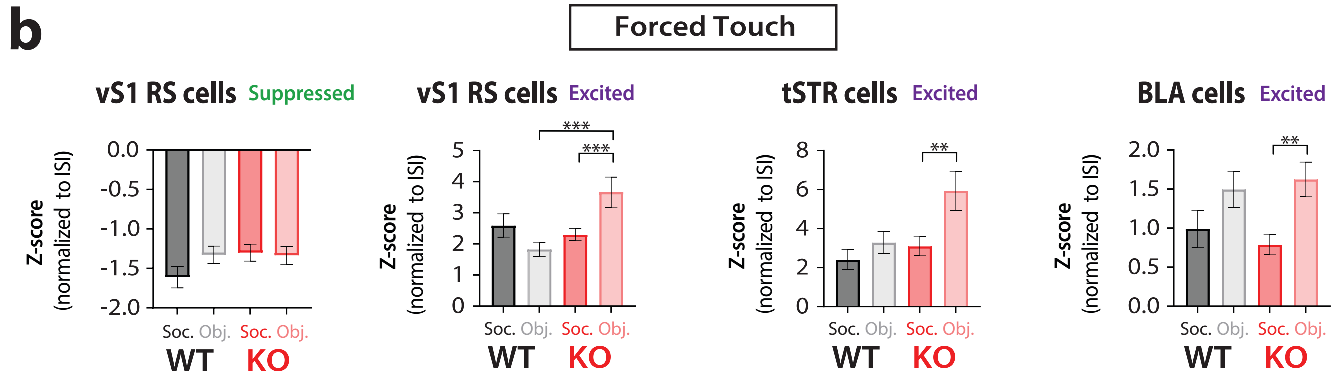

Supplementary Fig. 11: Clusters in vS1, tSTR and BLA are modulated differently by voluntary social and object touch in *Fmr1* KO mice.

a. Z-score firing rates, as a mean of all 40 presentations, for excited vS1, tSTR and BLA and suppressed vS1 cells during the 5 s period of touch for social and object voluntary touch in WT vs. *Fmr1* KO mice. ****p<0.0001, *p<0.05 for two-way ANOVA with Bonferroni's.

b. Z-score firing rates, as a mean of all 40 presentations, for excited vS1, tSTR and BLA and suppressed vS1 cells during the 5 s period of touch for social and object forced touch in WT vs. *Fmr1* KO mice. ****p<0.0001, ***p<0.001, **p<0.01 for two-way ANOVA with Bonferroni's.

For WT excited cells, vS1 n=227/220, tSTR 99/116, and BLA 81/79. For *Fmr1* KO excited cells, vS1 n=307/331, tSTR 178/202, and BLA 105/128. For *Fmr1* KO, suppressed vS1 n=77/110 (panels b-c). All data presented as mean ± SEM.

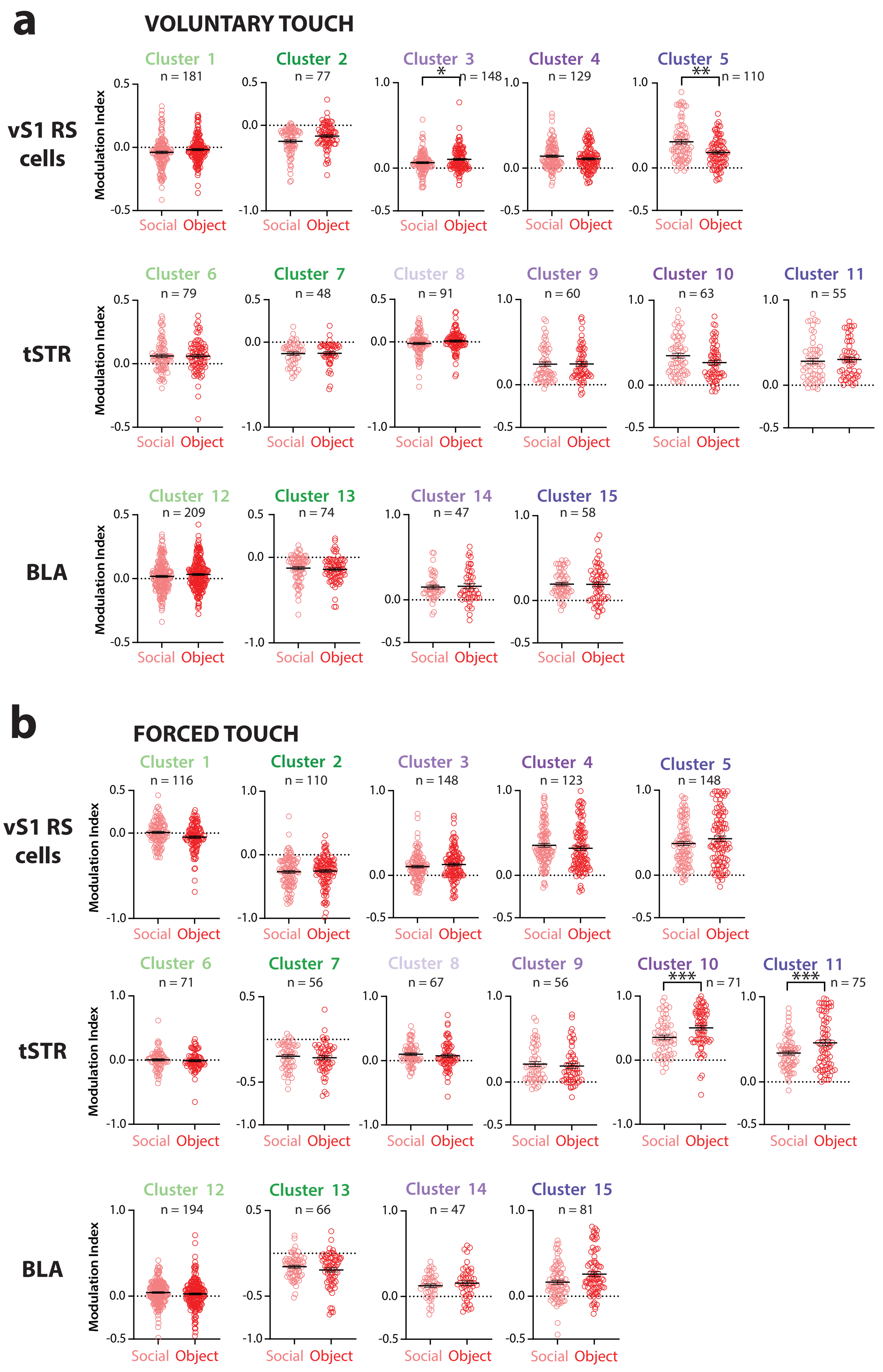

Relates to Fig. 6
Compare to Suppl. Fig. 6 in WT

Supplementary Fig. 12: Clusters in vS1, tSTR and BLA are modulated differently by voluntary social and object touch in *Fmr1* KO mice.

a. Modulation index of vS1 RS, tSTR and BLA cells in different clusters for social vs. object touch, during voluntary presentations, as an average of all 40 presentations. N denotes number of cells for the cluster.

b. Same as in panel *a*, but for forced touch. ****p<0.001, ***p<0.005, **p<0.01 for paired parametric t-test. Each symbol represents a single cell taken from across 10 *Fmr1* KO mice. Data presented as mean ± SEM.

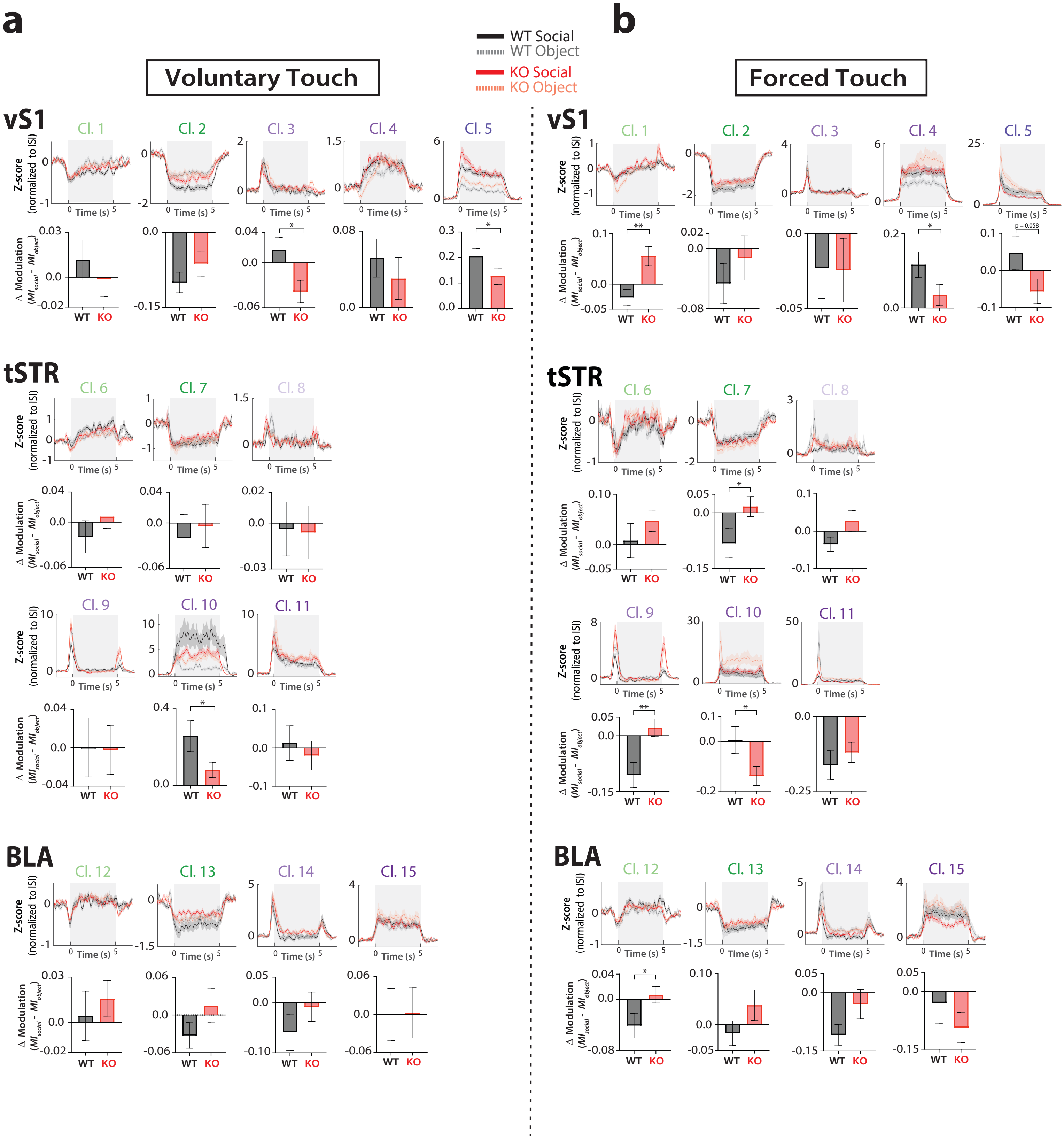

a
b
WT Social
WT Object
KO Social
KO Object
Voluntary Touch
Forced Touch
vS1
Cl. 1
Cl. 2
Cl. 3
Cl. 4
Cl. 5
tSTR
Cl. 6
Cl. 7
Cl. 8
Cl. 9
Cl. 10
Cl. 11
BLA
Cl. 12
Cl. 13
Cl. 14
Cl. 15
Z-score (normalized to ISI)
Time (s)
Δ Modulation (MI_{social} - MI_{object})
WT
KO
p = 0.058

Supplementary Fig. 13: Individual clusters in vS1, tSTR and BLA of *Fmr1* KO mice are modulated differently by social and object touch compared to WT mice.

a. Δ modulation ($MI_{social} - MI_{object}$) for voluntary social vs. object touch of cells in each cluster in vS1, tSTR and BLA of WT and *Fmr1* KO mice. *p<0.05, **p<0.01 for unpaired nonparametric or parametric t-test.

b. Δ modulation ($MI_{social} - MI_{object}$) for forced social vs. object touch of cells in each cluster in vS1, tSTR and BLA of WT and *Fmr1* KO mice. *p<0.05, **p<0.01 for unpaired nonparametric or parametric t-test.

Data presented as mean ± SEM.

a Decoding touch context (social. vs object) using firing rate (Hz) of cells from all mice

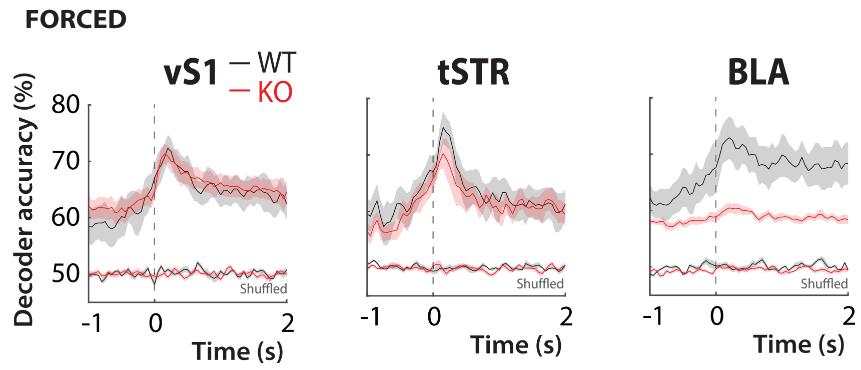

Relates to Fig. 6b

b Decoding touch context (social. vs object) using firing rate (Hz) of cells from only mice that were consistently running

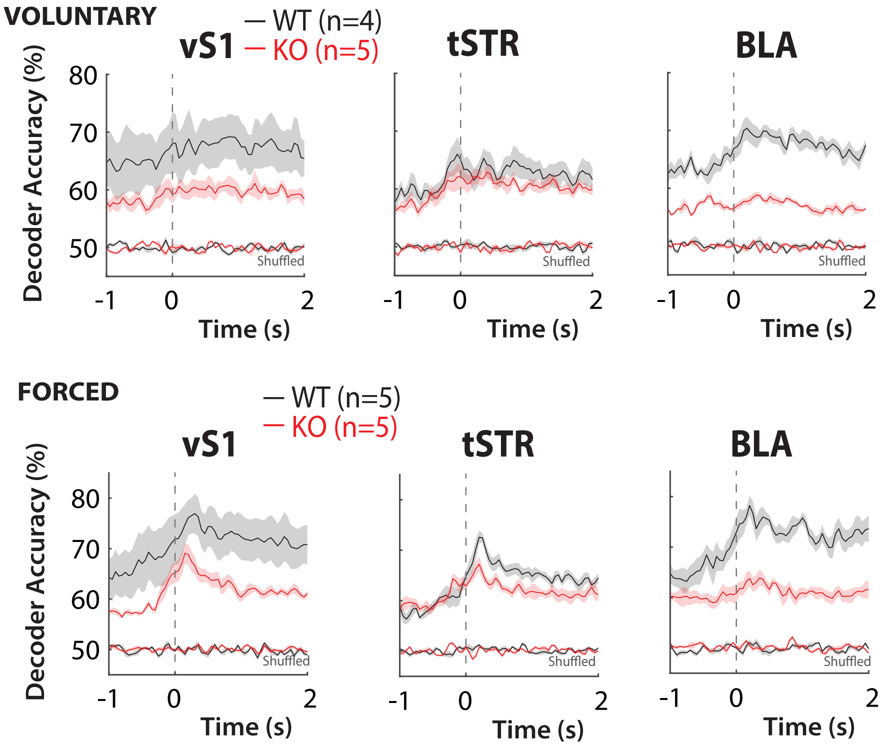

Relates to Fig. 6b

Supplementary Fig. 14: Decoding touch context (social vs. object) from mean neural activity is consistently worse in the BLA, and to an extent in vS1, of *Fmr1* KO mice.

a. Decoder accuracy for touch context, averaged across mice, based on activity of 10 randomly selected cells in vS1, tSTR and BLA (50 ms time bins) during the presentation period of forced touch (-1 to +2 s).

b. Decoder accuracy for touch context, averaged across a subset of WT and *Fmr1* KO mice that were consistently running throughout all presentations of social and object touch, based on activity of 10 randomly selected cells in vS1, tSTR and BLA (50 ms time bins) during the presentation period of voluntary and forced touch (-1 to +2 s).

Data presented as mean ± SEM.

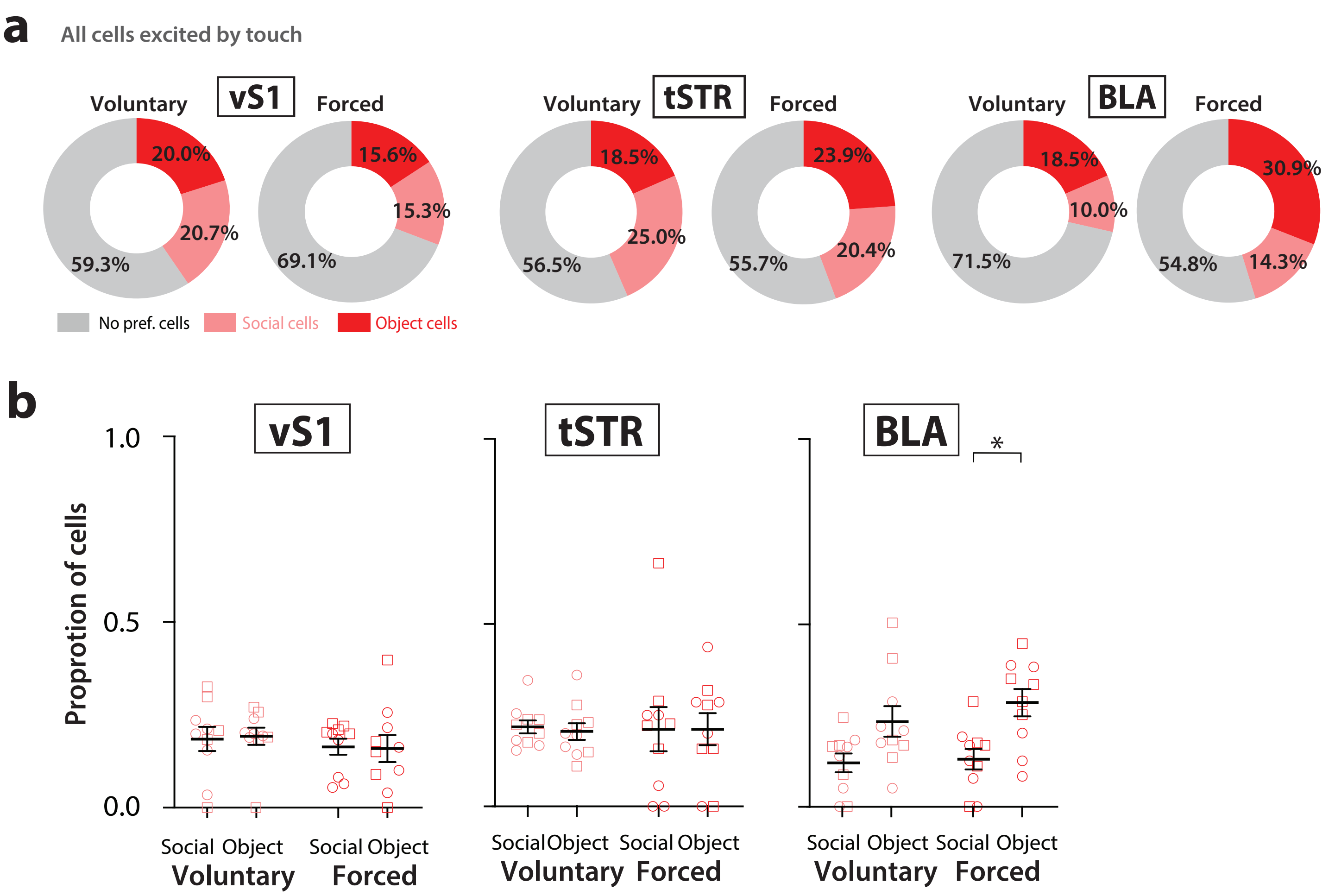

a
All cells excited by touch
vS1
Voluntary
Forced
20.0%
20.7%
59.3%
15.6%
15.3%
69.1%
tSTR
Voluntary
Forced
18.5%
25.0%
56.5%
23.9%
20.4%
55.7%
BLA
Voluntary
Forced
18.5%
10.0%
71.5%
30.9%
14.3%
54.8%
No pref. cells
Social cells
Object cells
b
Proprotion of cells
1.0
0.5
0.0
vS1
tSTR
BLA
*
Social Object Social Object
Voluntary Forced
Social Object Social Object
Voluntary Forced
Social Object Social Object
Voluntary Forced

Supplementary Fig. 15: Total proportion of social and object-preferring cells in vS1, tSTR and BLA of *Fmr1* KO.

a. Percentage of object-preferring and social-preferring cells in vS1, tSTR and BLA during voluntary and forced touch as total of all cells.

b. Proportion of object-preferring and social-preferring excited cells in vS1, tSTR and cells during voluntary and forced touch in *Fmr1* KO mice. Squares=males, circles=females. *$p<0.05$ for paired parametric t-test. N=8 WT and 10 *Fmr1* KO mice.

Data presented as mean ±SEM.

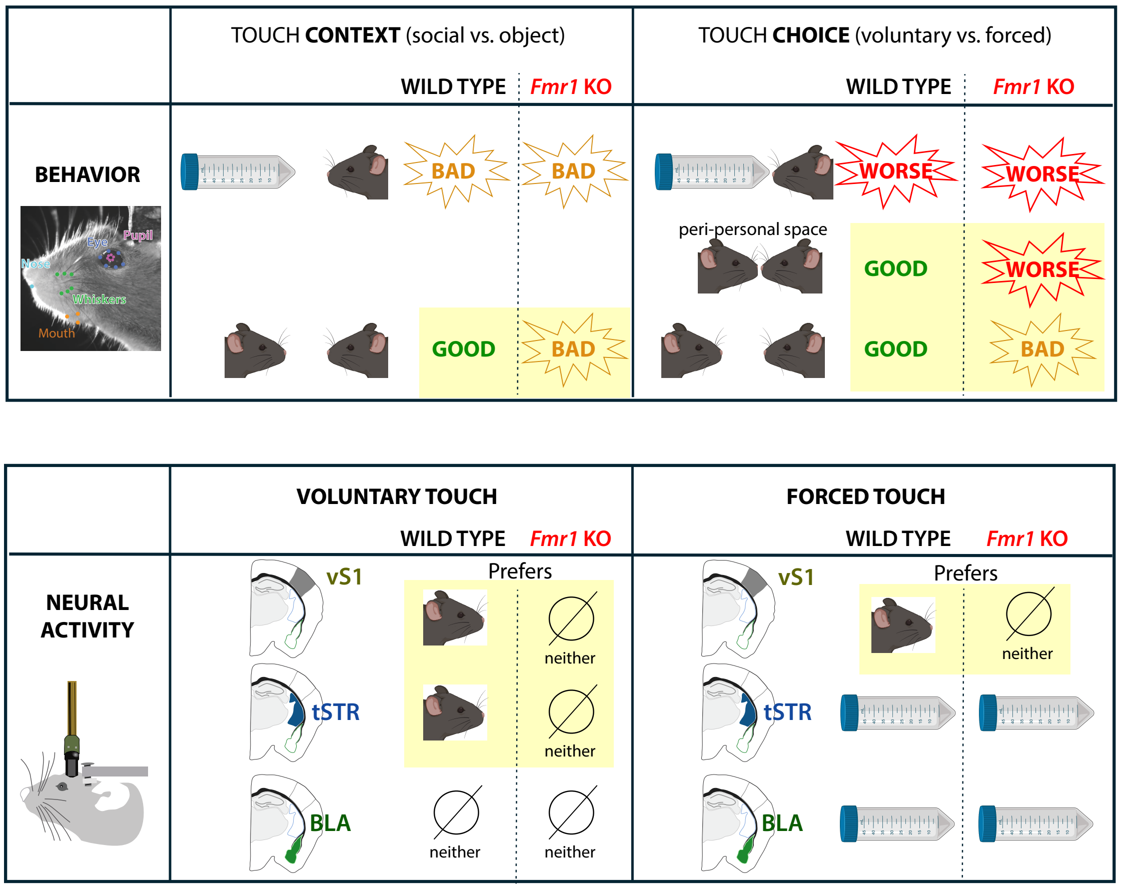

TOUCH CONTEXT (social vs. object)
TOUCH CHOICE (voluntary vs. forced)
WILD TYPE
Fmr1 KO
WILD TYPE
Fmr1 KO
BEHAVIOR
Pupil
Eye
Nose
Whiskers
Mouth
BAD
BAD
WORSE
WORSE
peri-personal space
GOOD
WORSE
GOOD
BAD
GOOD
BAD
VOLUNTARY TOUCH
FORCED TOUCH
WILD TYPE
Fmr1 KO
WILD TYPE
Fmr1 KO
NEURAL ACTIVITY
vS1
Prefers
neither
tSTR
neither
BLA
neither
neither
vS1
Prefers
neither
tSTR
BLA

Supplementary Fig. 16: Summary of main findings.

Here we summarize the main results related to behavioral responses (top) and neural activity (bottom). We compare how behaviors and neural activity differ according to differences in touch context (social versus object) and touch choice (voluntary versus forced interactions). For behavior, “good” means mice show minimal or no aversion; “bad” and “worse” means the mice experience increasing degrees of aversion. We also illustrate how responses of *Fmr1* KO mice differ from those of WT mice (note that yellow highlights underline the differences in behavior and neural activity between genotypes). Created in BioRender. Lim, K. (2025) https://BioRender.com/ppucaq3.

Figure Number	p-values for all two-way ANOVA tests
Fig. 4e	Brain region: Row-context, column-choice, row x column interaction<br>vS1: 0.282, 0.404, 0.733<br>tSTR: 0.473, 0.499, 0.030<br>BLA: 0.096, 0.094, 0.099
Fig. 5a-b	Behavior: Row-context, column-choice, row x column interaction<br>Running avoidance: 0.406, 0.651, 0.632<br>Aversive whisker protraction: 0.040, 0.004, 0.061<br>Orbital tightening: 0.229, 0.076, 0.762
Fig. 5c	Behavior: Row-genotype, column-choice, row x column interaction<br>AFEs: 0.003, 0.011, 0.314
Fig. 7d	Brain region: Row-regressors, column-genotype, row x column interaction<br>vS1: 0.003, 0.014, 0.934<br>tSTR: 0.161, 0.446, 0.875<br>BLA: 0.251, 0.059, 0.744
Suppl. Fig. 9a	Behavior: Row-context, column-choice, row x column interaction<br>Aversive whisker protraction: 0.051, 0.005, 0.075
Suppl. Fig. 10c	Touch choice & cluster group: Row-context, column-genotype, row x column interaction<br>Voluntary & vS1 suppressed: <0.001, 0.017, 0.263<br>Voluntary & vS1 excited: <0.001, 0.256, 0.025<br>Voluntary & tSTR excited: 0.004, 0.918, 0.074<br>Voluntary & BLA excited: 0.707, 0.841, 0.589<br><br>Forced & vS1 suppressed: 0.129, 0.032, 0.362<br>Forced & vS1 excited: 0.055, 0.016, 0.034<br>Forced & tSTR excited: <0.001, 0.422, 0.249<br>Forced & BLA excited: 0.005, 0.844, 0.613
Suppl. Fig. 11a-b	Touch choice & cluster group: Row-context, column-genotype, row x column interaction<br>Voluntary & vS1 suppressed: <0.001,0.093, 0.041<br>Voluntary & vS1 excited: <0.001, 0.042, 0.313<br>Voluntary & tSTR excited: 0.06, 0.337, 0.137<br>Voluntary & BLA excited: 0.244, 0.457, 0.124<br><br>Forced & vS1 suppressed: 0.272, 0.076, 0.166<br>Forced & vS1 excited: 0.423, 0.042, 0.005<br>Forced & tSTR excited: 0.004, 0.073, 0.234<br>Forced & BLA excited: <0.001, 0.886, 0.407
Suppl. Fig. 15b	Brain region: Row-context, column-choice, row x column interaction<br>vS1: 0.980, 0.277, 0.733<br>tSTR: 0.888, 0.995, 0.899<br>BLA: 0.002, 0.073, 0.643

Supplementary Table 1: Statistics for two-way ANOVA & Kruskal-Wallis test
