# Supplementary material for: A reduced ability to discriminate social from non-social touch at the circuit level may underlie social avoidance in autism
Source: Nat Commun. 2025 May 17;16:4600. doi: 10.1038/s41467-025-59852-6 (PMC12085590; doi:10.1038/s41467-025-59852-6)
Supplement: Supplementary file 2 — Reporting Summary [file 41467_2025_59852_MOESM2_ESM.pdf]

Corresponding author(s): *Double-anonymous peer review submissions: write DAPR and your manuscript number here instead of author names.*

Last updated by author(s): *YYYY-MM-DD*

Reporting Summary

Nature Portfolio wishes to improve the reproducibility of the work that we publish. This form provides structure for consistency and transparency in reporting. For further information on Nature Portfolio policies, see our Editorial Policies and the Editorial Policy Checklist.

Statistics

For all statistical analyses, confirm that the following items are present in the figure legend, table legend, main text, or Methods section.

n/a	Confirmed	
☐	☒	The exact sample size (*n*) for each experimental group/condition, given as a discrete number and unit of measurement
☐	☒	A statement on whether measurements were taken from distinct samples or whether the same sample was measured repeatedly
☐	☒	The statistical test(s) used AND whether they are one- or two-sided<br>*Only common tests should be described solely by name; describe more complex techniques in the Methods section.*
☐	☒	A description of all covariates tested
☐	☒	A description of any assumptions or corrections, such as tests of normality and adjustment for multiple comparisons
☐	☒	A full description of the statistical parameters including central tendency (e.g. means) or other basic estimates (e.g. regression coefficient) AND variation (e.g. standard deviation) or associated estimates of uncertainty (e.g. confidence intervals)
☐	☒	For null hypothesis testing, the test statistic (e.g. F, t, r) with confidence intervals, effect sizes, degrees of freedom and P value noted<br>*Give P values as exact values whenever suitable.*
☒	☐	For Bayesian analysis, information on the choice of priors and Markov chain Monte Carlo settings
☒	☐	For hierarchical and complex designs, identification of the appropriate level for tests and full reporting of outcomes
☐	☒	Estimates of effect sizes (e.g. Cohen's *d*, Pearson's *r*), indicating how they were calculated

Our web collection on statistics for biologists contains articles on many of the points above.

Software and code

Policy information about availability of computer code

Data collection	During the social touch behavioral assay and tactile defensiveness assay, electrophysiological recordings were performed using Neuropixels 1.0 acquisition hardware (Imec). The acquisition hardware was used in combination with PCI eXtensions for Instrumentation (PXI) hardware (PXIe-1071 chassis, PXIe-8381 remote control module and PXIe677 6341 I/O module for recording analog and digital inputs, National Instruments). SpikeGLX software was used to acquire data (https://github.com/billkarsh/SpikeGLX, HHMI/Janelia Research Campus).
Data analysis	Behavior and electrophysiological data was analyzed using custom written Matlab code as previously described (Chari, T. et al., J. Neurosci. 43 (43), 7158-7174 (2023), Rahmatullah, N. et al. J Neurosci. 48 (43), 8172-8188 (2023)) and available at https://github.com/porteralab and https://github.com/jcouto/pnc_spks. Quantification of TRAP labeling was analyzed using ImageJ. All statistical analyses was performed using GraphPad Prism.

For manuscripts utilizing custom algorithms or software that are central to the research but not yet described in published literature, software must be made available to editors and reviewers. We strongly encourage code deposition in a community repository (e.g. GitHub). See the Nature Portfolio guidelines for submitting code & software for further information.

Data

Policy information about availability of data

All manuscripts must include a data availability statement. This statement should provide the following information, where applicable:

- Accession codes, unique identifiers, or web links for publicly available datasets
- A description of any restrictions on data availability
- For clinical datasets or third party data, please ensure that the statement adheres to our policy

Data analyzed for this study has been deposited in FigShare. For electrophysiological data, see https://doi.org/10.6084/m9.figshare.28689119.v1. For behavioral data by trial, see https://doi.org/10.6084/m9.figshare.28689122.v1. For TRAP data, see https://doi.org/10.6084/m9.figshare.28689209.v3. The data generated in this study is also provided in a Source Data file for each figure.

Research involving human participants, their data, or biological material

Policy information about studies with human participants or human data. See also policy information about sex, gender (identity/presentation), and sexual orientation and race, ethnicity and racism.

Reporting on sex and gender	*Use the terms sex (biological attribute) and gender (shaped by social and cultural circumstances) carefully in order to avoid confusing both terms. Indicate if findings apply to only one sex or gender; describe whether sex and gender were considered in study design; whether sex and/or gender was determined based on self-reporting or assigned and methods used.*<br>*Provide in the source data disaggregated sex and gender data, where this information has been collected, and if consent has been obtained for sharing of individual-level data; provide overall numbers in this Reporting Summary. Please state if this information has not been collected.*<br>*Report sex- and gender-based analyses where performed, justify reasons for lack of sex- and gender-based analysis.*
Reporting on race, ethnicity, or other socially relevant groupings	*Please specify the socially constructed or socially relevant categorization variable(s) used in your manuscript and explain why they were used. Please note that such variables should not be used as proxies for other socially constructed/relevant variables (for example, race or ethnicity should not be used as a proxy for socioeconomic status).*<br>*Provide clear definitions of the relevant terms used, how they were provided (by the participants/respondents, the researchers, or third parties), and the method(s) used to classify people into the different categories (e.g. self-report, census or administrative data, social media data, etc.)*<br>*Please provide details about how you controlled for confounding variables in your analyses.*
Population characteristics	*Describe the covariate-relevant population characteristics of the human research participants (e.g. age, genotypic information, past and current diagnosis and treatment categories). If you filled out the behavioural & social sciences study design questions and have nothing to add here, write "See above."*
Recruitment	*Describe how participants were recruited. Outline any potential self-selection bias or other biases that may be present and how these are likely to impact results.*
Ethics oversight	*Identify the organization(s) that approved the study protocol.*

Note that full information on the approval of the study protocol must also be provided in the manuscript.

Field-specific reporting

Please select the one below that is the best fit for your research. If you are not sure, read the appropriate sections before making your selection.

☒ Life sciences ☐ Behavioural & social sciences ☐ Ecological, evolutionary & environmental sciences

For a reference copy of the document with all sections, see nature.com/documents/nr-reporting-summary-flat.pdf

Life sciences study design

All studies must disclose on these points even when the disclosure is negative.

Sample size	Sample sizes were not based on a priori power calculations but are consistent with other studies in the field using similar techniques, including our own (Chari, T. et al., J. Neurosci. 43, 7158-7174 (2023); He, C. X. et al., J. Neurosci. 37, 6475–6487 (2017); Mostany, R. et al., J. Neurosci. 30, 14116–14126 (2010); Goel, A. et al., Nat. Neurosci. 21, 1404–1411 (2018).
Data exclusions	Robust regression and outlier removal (ROUT) analysis was used to exclude outliers for data represented as individual mice. The standard deviation rule was used to exclude neurons with larges changes in baseline firing and neurons with large β weights in the linear encoding model for a given animal. One WT animal was excluded from behavioral decoding as videos were not synchronized across cameras.
Replication	Our study consisted of animals from at least two different litters for each genotype/group. We also tested animals in the social touch assay to reproduce the behavioral findings from our previous paper (Chari, T. et al., J. Neurosci. 43, 7158-7174 (2023).
Randomization	Randomization was performed by the experimenter to balance group size and sex as closely between groups as possible.

Blinding	Investigators were blinded to genotype of the animal (WT vs Fmr1 KO) for Neuropixels recordings. However, some Fmr1 KO mice can show hyperactivity compared to wt controls, which can be recognized by the experimenter. On very rare occasions Fmr1 KO mice manifested seizures, but those animals were excluded

Reporting for specific materials, systems and methods

We require information from authors about some types of materials, experimental systems and methods used in many studies. Here, indicate whether each material, system or method listed is relevant to your study. If you are not sure if a list item applies to your research, read the appropriate section before selecting a response.

Materials & experimental systems

n/a	Involved in the study
☒	☐ Antibodies
☒	☐ Eukaryotic cell lines
☒	☐ Palaeontology and archaeology
☐	☒ Animals and other organisms
☒	☐ Clinical data
☒	☐ Dual use research of concern
☒	☐ Plants

Methods

n/a	Involved in the study
☒	☐ ChIP-seq
☒	☐ Flow cytometry
☒	☐ MRI-based neuroimaging

Animals and other research organisms

Policy information about studies involving animals; ARRIVE guidelines recommended for reporting animal research, and Sex and Gender in Research

Laboratory animals	Adult male and female C5BL/6 mice at postnatal day 60-90 were used for all experiments. A cohort of adult mice (9 male and 8 female) were used for TRAP labeling of neurons activated by social/object touch. These so-called 'TRAP' mice were obtained by crossing Fos2A-iCreER/+ (TRAP2) (JAX line 021882) with R26Ai14/+ (Ai14) (JAX line 030323). A second cohort of mice (>20 g in weight) was used for electrophysiological recordings and were derived from the following mouse lines based on prior publications: wildtype (WT) B6J (JAX line 000664), Fmr1 KO (JAX line 003025). In total 9 WT (6 male and 3 female), and Fmr1 KO mice (6 male, and 4 female) were used for Neuropixels recordings.
Wild animals	No wild animals were used in this study.
Reporting on sex	We distinguished males from females across all figures showing data from each animal (squares depict males, circles depict females).
Field-collected samples	No field-collected samples were used in this study.
Ethics oversight	All experiments followed the U.S. National Institutes of Health guidelines for animal research under an animal use protocol (ARC #2007-035) approved by the Chancellor's Animal Research Committee and Office for Animal Research Oversight at the University of California, Los Angeles.

Note that full information on the approval of the study protocol must also be provided in the manuscript.

Plants

Seed stocks	*Report on the source of all seed stocks or other plant material used. If applicable, state the seed stock centre and catalogue number. If plant specimens were collected from the field, describe the collection location, date and sampling procedures.*
Novel plant genotypes	*Describe the methods by which all novel plant genotypes were produced. This includes those generated by transgenic approaches, gene editing, chemical/radiation-based mutagenesis and hybridization. For transgenic lines, describe the transformation method, the number of independent lines analyzed and the generation upon which experiments were performed. For gene-edited lines, describe the editor used, the endogenous sequence targeted for editing, the targeting guide RNA sequence (if applicable) and how the editor was applied.*
Authentication	*Describe any authentication procedures for each seed stock used or novel genotype generated. Describe any experiments used to assess the effect of a mutation and, where applicable, how potential secondary effects (e.g. second site T-DNA insertions, mosiacism, off-target gene editing) were examined.*
